# A relevance model of human sparse communication in cooperation

**DOI:** 10.3389/frobt.2025.1512099

**Published:** 2025-07-30

**Authors:** Kaiwen Jiang, Boxuan Jiang, Anahita Sadaghdar, Rebekah Limb, Tao Gao

**Affiliations:** ^1^ Department of Statistics and Probability, Michigan State University, East Lansing, MI, United States; ^2^ Department of Statistics and Data Science, University of California Los Angeles, Los Angeles, CA, United States; ^3^ Department of Psychology, University of California Los Angeles, Los Angeles, CA, United States; ^4^ Department of Communication, University of California Los Angeles, Los Angeles, CA, United States

**Keywords:** relevance, decision theory, theory of mind, POMDP, large language model, artificial intelligence

## Abstract

Human real-time communication creates a limitation on the flow of information, which requires the transfer of carefully chosen and condensed data in various situations. We introduce a model that explains how humans choose information for communication by utilizing the concept of “relevance” derived from decision-making theory and Theory of Mind (ToM). We evaluated the model by conducting experiments where human participants and an artificial intelligence (AI) agent assist each other to avoid multiple traps in a simulated navigation task. The relevance model accurately depicts how humans choose which trap to communicate. It also outperforms GPT-4, which participates in the same task by responding to prompts that describe the game settings and rules. Furthermore, we demonstrated that when humans received assisting information from an AI agent, they achieved a much higher performance and gave higher ratings to the AI when it utilized the relevance model compared to a heuristic model. Together, these findings provide compelling evidence that a relevance model rooted in decision theory and ToM can effectively capture the sparse and spontaneous nature of human communication.

## 1 Introduction

In a football game, the receiver catches the ball. Despite three opponent defenders rapidly approaching him, he accelerates without noticing them. Within that split second, you can only alert him about one of the opponents. How do you make that choice in fast and instantaneous communication?

Living in a fast-paced modern world, we constantly face an overload of information while needing to make quick decisions of what to communicate. This necessity for rapid decision-making applies to contexts as diverse as emergency room operations, stock trading, and professional kitchens. In these domains, communication must be swift and effective, conveying substantial information efficiently.

But how do humans manage to distill complex information into concise, impromptu communicative signals? How do we determine what information to share? In this paper, we use pointing—a remarkably simple form of communication—as a case study to investigate how humans swiftly and effectively choose which information to communicate.

Pointing has been extensively studied within the fields of cognitive science and developmental psychology. Notably in developmental psychology, humans develop pointing even prior to the acquisition of language. For instance, infants have been shown to understand the implications of pointing from contextual cues and use pointing at 12 months old (Tomasello, 2010). Toddlers aged 18–36 months even point in consideration of others’ perspectives, exhibiting greater amounts of pointing when their partner’s view is obstructed (Franco and Gagliano, 2001).

Additionally, Misyak et al. (2016) studies human adult pointing behavior to demonstrate that human pointing is extremely overloaded and indirect. The same signal can have multiple different meanings and these meanings can communicate something beyond the object’s visual features, such as what to do with the referent and why the pointer employed such a gesture.

Pointing is overloaded, indirect, and sparse. How can humans accurately interpret it in a mere instant? This question may be answered by the interaction of humans and their surrounding environment: how we act in the environment imposes a critical constraint on the interpretation of pointing signals. From the perspective of decision theory, if we take upon the widely accepted assumption of human rationality, it is imperative for one to perform pointing signals that facilitate actions which lead to achieving the highest utility in the most efficient manner possible. For example, when a chef points to the cookware cabinet, the sous chef may not know what cookware the chef is asking for. However, in the context of making a salad, it is more likely that the chef is asking for a knife, while in the context of making a souffle, the chef is more likely to ask for a mixer. The meaning of the same pointing gesture can be dynamically determined by the task involving the sender and interpreter. Therefore, the richness and flexibility does not arise from the gesture itself, but from the variety of contextual constraints induced by tasks. In turn, capturing the decision making aspect of the task, estimating the utility of what has been done and what could be done, is the key to deciphering pointing.

What bridges the communication and contexts is a concept called relevance, which is widely studied in the field of linguistics. Grice (1975) introduced the maxim of relevance, asserting that speakers must provide information that is relevant to the current conversation if they wish for listeners to correctly understand their meaning. Similarly, listeners can successfully understand the intended meaning when they assume the speaker is adhering to this maxim. In Grice’s definition, a sentence is relevant if its meaning continues the dialogue. For example, if one asks where the scissors are and receives the response, “I cut paper in my room.” Assuming this response is relevant, it must continues the conversation even though it does not mention scissors at all. To afford the continuation of the conversation, the response must mean I cut paper with the scissors in my room, so we may find the scissors in the respondent’s room. Wilson and Sperber (2006) added that relevance can be assessed in terms of cognitive effect and processing effort. In their definition of relevance, relevant information helps the listener by creating a worthwhile difference in their understanding of the world. Notably so far, the discussions of relevance in linguistics focus primarily on the sentences in conversations. We aim to extend their work to non-verbal overloaded communication by incorporating components like Theory of Mind (ToM) and task-planning. We will define a sequential decision-making task with two agents: one helper who communicates with one actor only through pointing.

Recently, decision-theory is increasingly incorporated into communication studies. The rational speech act (RSA) model (Frank and Goodman, 2012), for instance, is a linguistic model developed from reference games, in which one attempts to find a target among distractors based on a description. The RSA model assigns each utterance a utility based on how much it can direct the listener’s belief towards the true target. Extending this reference game to decision-making, relevance emerges as an utterance’s ability to increase the listener’s reward. Studies on pedagogy reveal that individuals meticulously select their instructions by considering its impact on the listener’s performance and belief (Sumers et al., 2023a; Ho et al., 2016). Another study shows the proficiency of robot assistants in providing the most helpful–even if somewhat distorted–observations to aid task-solving (Reddy et al., 2021). Another line of work demonstrates on basing signal generation and inference of extremely overloaded signals. Interpreting a helper’s signals by assuming that the helper is trying to be maximally helpful significantly improves agent’s performance (Jiang et al., 2022). To further our understanding of relevance, we intend to build a relevance model-based decision theory and ToM that can capture the essence of human communication and support human-AI interaction.

The approach of modeling human decision-making through utility calculations has a long-standing tradition, with prospect theory being one notable example (de Souza et al., 2020). To further model how agents interact with their environment over time, sequential decision-making frameworks such as Markov decision process (MDP) are commonly used (Knox and Stone, 2012; Levin et al., 2002). When focusing on decisions driven by the agent’s internal mental states, it’s important to incorporate the agent’s beliefs and perception of information. In contexts like human navigation (Stankiewicz et al., 2006) and inverse reinforcement learning (Baker et al., 2011), where agents typically have access to only partial information, the partially observable Markov decision process (POMDP) framework is frequently employed. POMDPs are well-suited for capturing both human and autonomous robot behavior (Kurniawati and Yadav, 2016), making them a valuable tool in human-robot interaction (You et al., 2023; Singh et al., 2022; Nikolaidis et al., 2017; Wang et al., 2016; Nikolaidis et al., 2015; Lam and Sastry, 2014; Taha et al., 2011), autonomous driving (Qiu et al., 2020), and communication between humans (Ho et al., 2016) or between human and robot (Le Guillou et al., 2023). As an example, You et al. (2023) designed a POMDP-based robotic system to predict the human collaborator’s action and coordinate in a dual-agent task. Multi-agent POMDP models such as interactive POMDP (Gmytrasiewicz and Doshi, 2005) and decentralized POMDP (Oliehoek and Amato, 2016) further study the collaboration and communication between agents (Gmytrasiewicz and Adhikari, 2019).

Even though the POMDP model remarkably succeeded in modeling human decision-making in partially observable environments, it is not our main objective to build robotic systems to physically interact with humans and the environment, like in (You et al., 2023). Instead, we focus on modeling how humans select and interpret communicative signals, which only changes the mind of the agents. In the following section, we propose a utility-based relevance framework. With this objective, two crucial components, action prediction and action evaluation, depend on utility computed via POMDP in this study. Nevertheless, POMDP is not indispensable and may be substituted with alternative agent models capable of utility estimation. In our experiments, POMDP is adopted to model the decisions made by the player who physically changes the environment, but not to model the signal selection of the helper as they only change the player’s mind. Also with our objective, we measure the consistency of the predictions by our model with human behavior, in addition to the joint reward gained by the collaboration between human and agent.

## 2 Model

**FIGURE 1:**
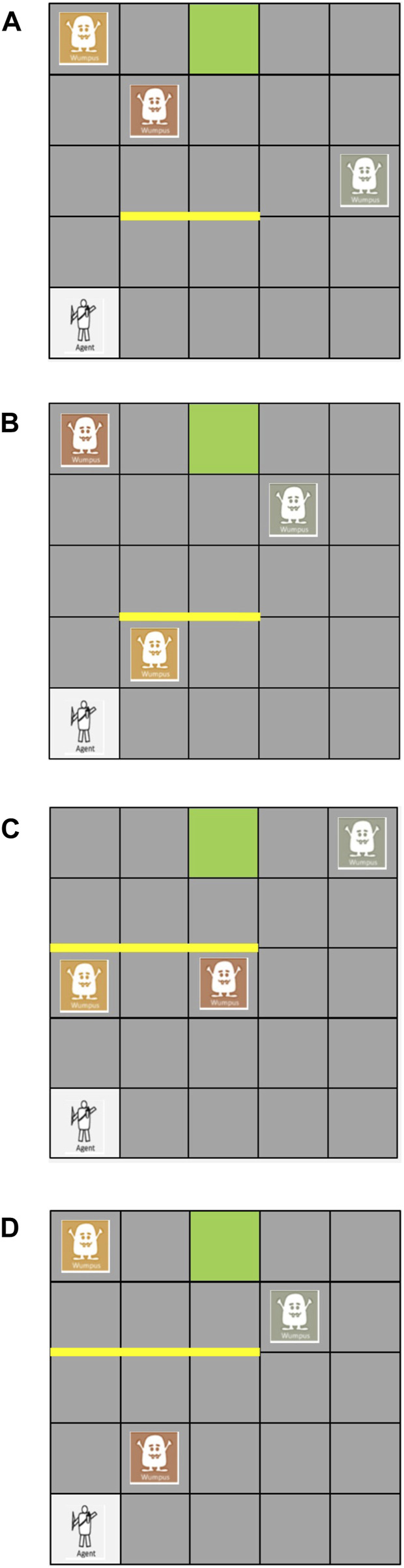
4 of the maps used in the experiments.

We design a game inspired by the impromptu and sparse communication in the football example in the introduction. In this game, a player navigates the map from a starting point to a goal. We design 5 by 5 maps with examples shown in Figure 1. As we assume a small cost of 1 for each step coupled with a substantial reward of 100 upon reaching the goal, the player wants to reach the goal as fast as possible.

Three static monsters are randomly distributed in the 23 tiles other than the start and the goal. The player’s objective is to avoid the monsters, because a penalty of 100 is incurred if the player steps into each monster. Stepping into a monster only results in a penalty and does not end the game. Importantly, the locations of the monsters are unknown to and cannot be perceived by the player. This lack of information is designed to motivate communication.

While the player is not aware of the locations of the monsters or able to take any actions to observe them, a helper who knows the locations of the monsters tries to assist the player. To highlight the sparsity of human communication, the helper can only point to one monster on the map when the player is at the starting point.

In this immediate and perilous situation that requires on-the-spot communication, the information communicated is far less than what is needed. We build a model for relevance and want to test whether the most relevant signal predicted by the model is consistent with the choice of the helper.

With the task as a running example, we define our relevance model. We start from the basics of modeling single-agent decision-making with a POMDP, then build a model of two-agent communication on top of them.

We model the player as a POMDP agent. In a POMDP, the world is defined as a state s . In our example, it is a tuple containing the location of the player himself and the three locations of the monsters. The agent also has a set of actions, which are moving left, right, up or down in our game. After taking an action a , the agent transition to the next state $s^{\prime }$ with a probability modeled by a transition model $P\left(s^{\prime }|s,a\right)$ while receiving a reward $R\left(s,a,s^{\prime }\right)$ . In this game, the player moves to an adjacent tile in the chosen direction unless the move would result in hitting a wall, in which case the player remains stationary. The reward structure is based on monster positions: each move costs 1 point; reaching the goal yields 100 points and ends the game; and colliding with a monster results in a 100-point penalty.

Utility is defined as a function that represents the desirability of a state. In our example, the desirability of a state is not only determined by its utility right now, but also the future consequences of actions the agent would take from this state. For an action a in a state s , the utility can be written as Q(s,a) . A completely rational agent will take the action with the highest utility. Thus, the value of the state will be the maximum utility of the state under all possible actions $V\left(s\right)=\backslash max_{a}Q\left(s,a\right)$ . In an optimal MDP policy, the agents take the action with the highest utility $a*=\arg\max_{a}Q\left(s,a\right)$ . However, to model the variability in human decision-making, we use a Boltzmann (or softmax) policy, which is commonly applied in studies of human decision-making (Baker et al., 2011; Jara-Ettinger et al., 2016):

$$P\left(a|s\right)\propto exp\left\{\alpha Q\left(s,a\right)\right\},$$ (1)

where α is a parameter controlling rationality, usually large. If α is higher, the agent is more strict in taking the action with the highest reward.

When the agent does not know the exact state, like the player in the game, it maintains a belief, a probability distribution over all possible states (Kaelbling et al., 1998). We can use b(s) to represent the probability of the current state being s according to belief b . In our task, the belief is a discrete probability distribution over all possible tuples of monster locations and self-location. As an example, for a player at the starting point, the 3 monsters will be located in the other 23 tiles, resulting in $\left({\;}_{\;3}^{\;23}\right)$ possible states. Assuming that the player has no knowledge of the monster locations, we can apply a flat prior: the probability will be $1/\left({\;}_{\;3}^{\;23}\right)$ for each state. If the player knows the locations of the monsters $m_{1}$ , $m_{2}$ , $m_{3}$ , and his location $s_{p}$ , then the state can be represented by a tuple $\left(s_{p},m_{1},m_{2},m_{3}\right)$ . His belief is $\delta \left(s_{p},m_{1},m_{2},m_{3}\right)$ , where δ is the Dirac delta function. After the agent taking each action, it receives an observation o from the environment, which in a Bayesian way updates its belief with a likelihood function $P\left(o|s^{\prime },a\right)$ . The player in our game does not receive any observation from the environment. The only change of its belief results from the helper’s communication of which tile seats a monster.

Unknowing of the world state, an agent makes decisions based on its belief. We can extend the definitions of Q and V to beliefs. In POMDP, the value of a belief can be defined as $V\left(b\right)=max_{a}Q\left(b,a\right)$ , while the utility of an action at a certain belief is assessed by considering what observations may result from the actions, and what could be the belief at the next time step. To solve the POMDP is to find the best strategy in a POMDP problem. The exact solution for a POMDP is hard to achieve, however it is possible to compute policies using approximate POMDP solvers such as the ones proposed by Pineau et al. (2003), Spaan and Vlassis (2005), Kurniawati et al. (2009), Silver and Veness (2010), Smith and Simmons (2012). Since the POMDP agent does not receive any observation in our specific game, we use a special case of POMDP, the QMDP to solve for the optimal policy (Littman et al., 1995). In QMDP, the belief-action utility function is the state-action utility weighted the agent’s belief

$$Q\left(b,a\right)=\underset{s}{\sum }Q\left(s,a\right)P\left(s|b\right).$$ (2)

Although Equation 2 and the QMDP approximation only hold for special POMDPs with no observations, our following formulation can be applied to generic POMDP problems with observations incorporated, with Q(b,a) approximated by a POMDP solver.

For a given policy π(a)=P(a|π) , the value of a belief under the policy is $V_{\pi }\left(b\right)=\sum_{a}Q\left(b,a\right)P\left(a|\pi \right)$ . The value of a belief is the maximum utility of the belief under all possible actions $V^{*}\left(b\right)=\max_{a}Q\left(b,a\right)$ . In our task, if the player at position (1, 3) is certain that a monster is at (2, 3), then his belief is $1/\left({\;}_{\;2}^{\;22}\right)$ for each state with (2, 3) as a monster and (1, 3) as self-location. With a goal at (2, 4), he will calculate the utility of the action of going to (2, 3) then (2, 4) to be Q=−100+100−2 , while the utility of the action of going to (1, 4) then (2, 4) to be $Q=\left({\;}_{\;1}^{\;21}\right)/\left({\;}_{\;2}^{\;22}\right)\left(-100\right)+100-2$ . A rational player will be more likely to choose the second path with an adaptation of Equation 1,

$$P\left(a|b\right)\propto \exp\left\{\alpha Q\left(b,a\right)\right\}.$$ (3)

We broaden our decision-making approach to encompass cooperative scenarios involving two agents, where communication plays a crucial role. Initially, we establish a belief system that takes into account not only the physical state of the world but also the beliefs held by the other agent. In our task, the player lacks knowledge of both the physical state and the helper’s belief. Therefore, the player’s belief $b_{P}$ can be represented through a joint probability distribution of s and the helper’s belief $b_{H}$ . We call this an interactive state IS , as in interactive-POMDP (Gmytrasiewicz and Doshi, 2005). $IS_{P}=\left(s,b_{H}\right)$ . In our task, the helper knows the world s* and the player’s the belief $b_{P}$ , so her belief is $P\left(IS_{H}\right)=\delta \left(s*,b_{P}\right)$ . The player’s belief $b_{P}$ is a flat prior on both the physical state and the helper’s belief. However, since he knows that the helper has full knowledge of the physical state, the helper’s belief and the physical state are not independent, but should have the constraint that the helper’s belief reflects the true state $b_{H}=\delta \left(s\right)$ . Therefore, his belief is $P\left(IS_{P}\right)=P\left(s,b_{H}|b_{P}\right)=1/\left({\;}_{\;3}^{\;23}\right)I\left(b_{H}=\delta \left(s\right)\right)$ . His belief about the state is the marginal distribution $P\left(s|b_{P}\right)=\sum_{b_{H}}P\left(s,b_{H}|b_{P}\right)$ .

To efficiently help the player in the game, the helper needs to predict and evaluate the player’s future actions. The helper estimates the player’s belief, assumes his rationality, and predicts his actions. For example, in Figure 1A, the helper is aware that the player does not know the monster is at (0, 4). She can predict his action to be moving up to the top then right, and the consequence to be hitting the monster. This prediction is a classic ToM application. The key is to use the player’s belief, not use the helper’s belief. Adapting from Equation 3, the helper can predict the player’s actions

$$P\left(a_{P}|b_{P}\right)\propto \exp\left\{\alpha Q\left(b_{P},a_{P}\right)\right\}$$ (4)

After predicting the player’s actions and consequences, the helper is free to use her own belief to evaluate these consequences. By adapting Equation 2, the helper can evaluate the player’s action $a_{P}$ with her own belief $b_{H}$ ,

$$Q\left(b_{H},a_{P}\right)=\underset{s}{\sum }Q\left(s,a_{P}\right)P\left(s|b_{H}\right).$$ (5)

If she predicts that the player is stepping into a monster, the helper will think the player is in a bad shape. She can then simulate what would happen if the player knew about the monster. If the player can avoid because of the access to this knowledge, then the helper’s knowledge is relevant as it improves the player’s wellbeing.

Combining the action prediction (Equation 4) and evaluation (Equation 5) steps, if the helper can predict the player’s actions $a_{P}$ from his belief $b_{P}$ , then she can evaluate $b_{P}$ with her own belief $b_{H}$ .

$$V\left(b_{P}|b_{H}\right)=\underset{a_{P}}{\sum }Q\left(b_{H},a_{P}\right)P\left(a_{P}|b_{P}\right).$$ (6)

Equation 6 is important because it shows that the helper can evaluate the players belief based on her own belief of the world. In our task, the helper always knows better of the world than the player, so she can always do better if she were in the player’s position. The difference between the helper’s evaluation of the player’s belief and her own belief is the motivation for the helper to communicate with the player. Only when there is a difference, the helper sees herself as capable of saying anything relevant. Therefore, we can define this difference in utility as the relevance of the helper’s belief to the player’s belief.

$$Relevance\left(b_{P}|b_{H}\right)=V\left(b_{H}|b_{H}\right)-V\left(b_{P}|b_{H}\right).$$ (7)

The relevance of the helper’s belief (Equation 7) is the maximum amount they can help. In real-life, while a helper may want to pass along excessive information to a person, she can only change the person’s belief with an act of sparse communication.

Defining the relevance of a belief is critical, but not enough. In real world, we cannot directly copy our belief to the other. The change of belief must go through the interpretation of an utterance. That’s why we need to define the relevance of an utterance. For an utterance u , we represent the belief of its receiver to be updated to $b_{P}\left(u\right)$ from the original belief $b_{P}$ . In our task, three utterances are available: each of the three monsters. The interpretation is clear: a monster is there. The improvement in utility caused by the utterance is

$$Relevance\left(u|b_{P},b_{H}\right)=V\left(b_{P}\left(u\right)|b_{H}\right)-V\left(b_{P}|b_{H}\right)$$ (8)

In Figure 1D, if the helper points to the green monster, it is relevant because it helps improve the player’s utility. In contrast, pointing to the orange monster is not relevant because it does not improve the player’s utility at all. With relevance of a communicative signal calculated with Equation 8, we predict that the rational helper will most likely point to the most relevant monster with the probability

$$P\left(u|b_{P},b_{H}\right)\propto \exp\left\{\alpha Relevance\left(u|b_{P},b_{H}\right)\right\}.$$ (9)

## 3 Experiments

### 3.1 Measure human navigation prior through inverse reinforcement learning

In order to evaluate the effectiveness of our relevance model, we aim to use it to predict the relevance-based choices of monsters to point, and compare our predictions with the choices of human participants. First, we want to make the decision-making process similar to human participants, so that we can evaluate the beliefs and utterances accurately.

For reinforcement learning agents like a rational agent employing an MDP solver, actions are selected with the same probability if they provide the same future expected values Q . Reflected in our maps, it means if there are two moves giving you the same Q , these agents will choose them with the same probability regardless of their directions. However, by observing human walking and playing video games, we noted that humans do not always choose between these trajectories evenly. Individuals tend to continue moving in the same direction as their initial movement (Arechavaleta et al., 2008). Also, they often opt for actions that align geometrically with the goal, choosing movements that bring them closer to the destination.

**FIGURE 2:**
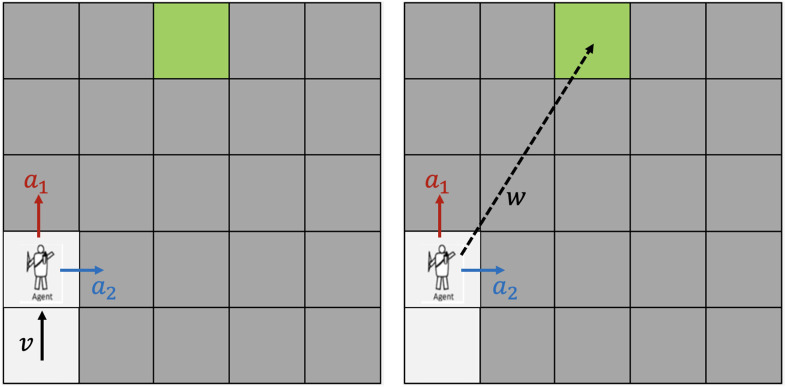
A frame in display of Experiment 1: after participant selects monster at position (2, 3) and completes helpfulness self-rating, the player figure moves to the goal.

To capture human preference of moving directions, we utilize the concept of feature expectation within inverse reinforcement learning. This involves formulating the reward as a linear combination of various features associated with the state (Abbeel and Ng, 2004; Bobu et al., 2020). Apart from the initial reward function R , we introduce two additional features. The first feature calculates the angle between the intended action a and the previous movement direction v , $r_{v}=cos\left(a,v\right)$ . The second feature computes the angle between the intended action a and a vector w extending from the current position to the goal, $r_{w}=cos\left(a,w\right)$ . We introduce two additional features as shown in Figure 2.

We undertook a pilot experiment to determine the appropriate weights for these two features in influencing human decision-making during navigation. We recruited five participants to navigate the maps in our experiment (shown in Figure 1) but without the monsters (five unique maps). For each trajectory j , we calculate its reward from the environment $R\left(j\right)=\sum_{t}r_{t}$ , the reward from following previous direction $v_{j}=\sum_{t}r\left(v,t\right)$ , and the reward from moving towards the goal $w_{j}=\sum_{t}r\left(w,t\right)$ . We fit a logit model $\log\frac{P\left(j\right)}{1-P\left(j\right)}=R\left(j\right)+\alpha_{1}v_{j}+\alpha_{2}w_{j}$ to learn the reward function for the agent (Train, 2009). With the trajectory collected, we estimate the parameters $\alpha_{1}=2.497$ , $\alpha_{2}=5.077$ . The reward $R+\alpha_{1}r\left(v,t\right)+\alpha_{3}r\left(w,t\right)$ is only used to predict humans’ navigation policy. When evaluating the policies, we use the original reward R from the environment.

### 3.2 Experiment 1

We assigned participants as the helper in our task, while the player was controlled by a rational AI with QMDP-based approximation (see Equation 2) as policy solution for the POMDP. Our objective is to assess whether a relevance model can effectively predict the human selection of which monster to communicate within a map. We measure the correlation between the model’s predictions and the actual choices made by humans.

#### 3.2.1 Participants

20 undergraduate and graduate students participated in this online study and were compensated with 5 dollar gift cards. Since we newly designed this type of experiment, we do not have a clear estimation of effect size, so we choose 20 as the participant size, which is commonly adopted in lab-based experiments.

#### 3.2.2 Stimuli

We generated a set of 14 distinct maps, each of which can be horizontally mirrored, resulting in a total of 28 maps. Example maps are shown in Figure 1. Among the 14 original maps, a player figure representing the player’s starting position is located at position (0, 0). In the mirrored maps, the player’s starting position is shifted to (4, 0). To introduce more diverse policies, we incorporated horizontal or vertical barriers in 9 out of the 14 maps. These barriers prevent the player from moving through them, forcing the participants to take a detour.

The 14 maps were manually crafted with the goal of maximizing the variation of relevance in each map, guided by the following principles. First, we ensured that there is always a monster positioned off the shortest paths, like the green monster in Figures 1A–C, and the orange monster in Figure 1D. This monster’s relevance is intentionally set to be very low: rational agent will not pass through this location. Second, we strategically placed some monsters on a cell that multiple shortest paths pass through, sometimes encompassing all the shortest paths. This monster will have a very high relevance, since the consequences of not knowing its location are undesirable: the player has a high probability of running into this monster.

Third, we introduce the blocking mechanism to show that relevance is not about whether the monster is on a shortest path or not, but on the location-based utility calculation. This manipulation involved an arrangement requiring all the shortest paths passing through the first monster to also pass through the second monster, like the monsters in Figure 1D. On its own, the red monster might have high relevance. However, since all the paths through the red monster (referred to as blocked) must go through the green monster (referred to as blocking), only knowing the red monster does not help the player too much: he will still run into the green monster. In contrast, knowing the green monster helps very much. These two monsters are both on many shortest paths, but the relevance is dramatically different. We assume that to thrive in this type of map, agents need the capacity of forward simulation. They need to think “what would happen if I point to that monster instead?” to point to the blocking monster instead of the blocked one.

**FIGURE 3:**
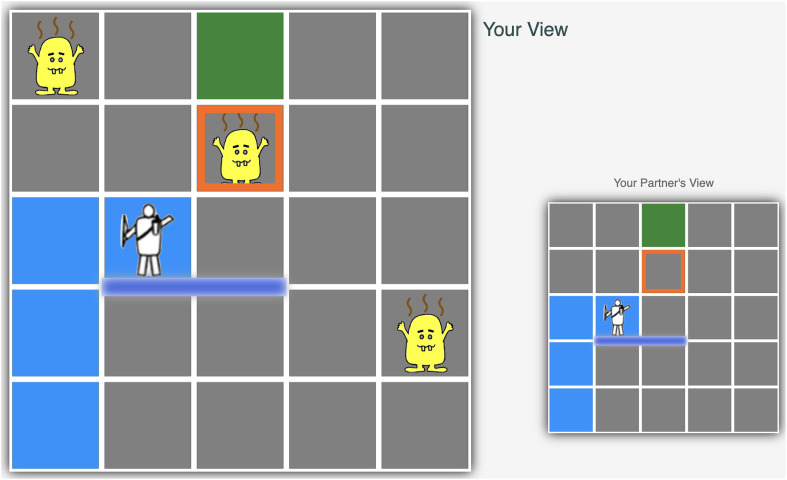
Features used to model participants’ innate reward when selecting trajectory. Left: participants tend to choose $a_{1}$ which aligns with momentum v ; Right: participants tend to choose $a_{1}$ which directs closer to the goal.

The colors used in the examples are solely for illustration. It is important to note that the monsters are all displayed as the same color in the actual experiment, as in Figure 3.

#### 3.2.3 Design and procedure

Participants joined the experiment by accessing a link on their personal computers. The use of mobile phones and tablets was prohibited for this purpose.

Upon joining the experiment, participants were provided with a tutorial that includes an informed consent document. Then, they were given a comprehension quiz to ensure they understood the instructions properly. Prior to the formal trials, participants went through six practice trials. The practice phase included working with three maps and their respective mirror images all presented in a random order.

After completing the practice trials, participants proceeded to the main experiment which comprised of 28 trials. During each trial, the participants were presented with two maps side by side (Figure 2). A large map was displayed on the left side, showing the perspective of the helper (themselves) and revealing the locations of the monsters. The maps are displayed with a size of $8.5\times 8.5{\text{cm}}^{2}$ . In contrast, the small map on the right provided the player’s view for the participant’s reference. It did not show the locations of the monsters. The small maps are displayed with a size of $4.5\times 4.5{\text{cm}}^{2}$ . The trials were presented to each participant in a random order, ensuring that the original and mirrored versions of the same map were not shown in two consecutive trials. During the trials, participants need to assist their partner. They can choose to highlight a monster on the large map by double-clicking on the selected monster. The participants’ choice was then recorded.

After the participants confirmed their choice of highlighted monster, a nine-point Likert scale appeared on the screen. This scale prompts participants to rate the perceived helpfulness of their signal, ranging from 1 (not helpful) to 9 (very helpful). The rating reflects the participant’s perception of the helpfulness of the signal, the signal’s expected effect on the player, rather than the specific effect in this trial. Upon receiving the highlighted location from the participant, the AI player updates its belief, treating the marked location as a monster. It then updates its policy based on this new information and proceeds to navigate the map accordingly. The navigation route was visually presented to the participants as an animation. Once the player successfully reached the goal, a feedback box appeared on the screen. This feedback included details about the partner’s performance, such as the number of steps the player took, the number of monsters they encountered, and the score they received. After reviewing the feedback, participants proceeded to the next trial.

The participants took an exit survey after completing all the trials. The exit survey is an open question for the participant’s strategy and the opinion on the experiment.

#### 3.2.4 Results

**FIGURE 4:**
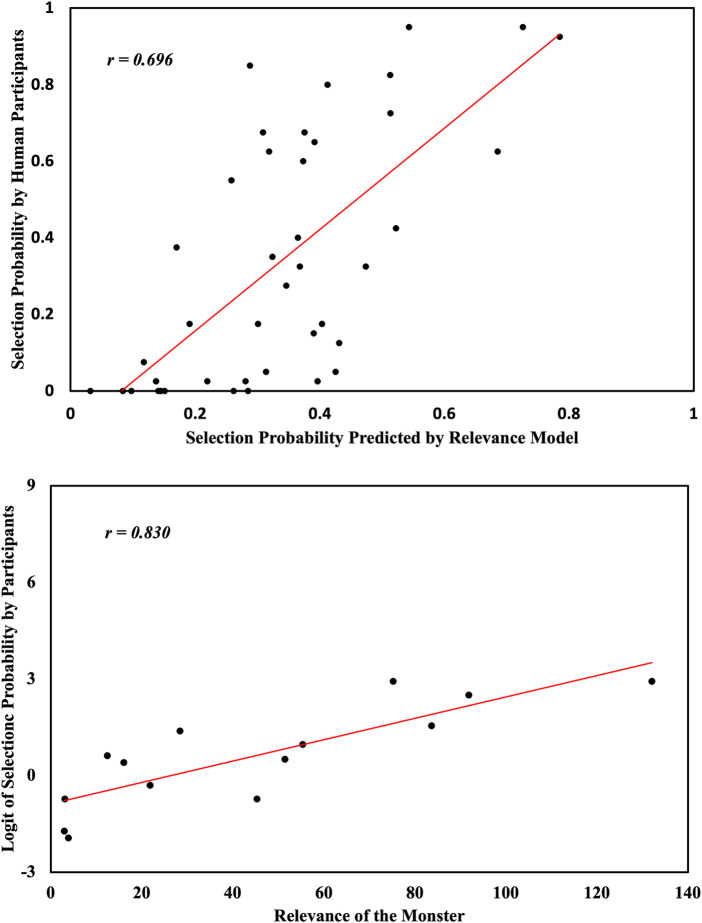
Our relevance model can predict human participants’ choices. Top: Predicted probability from relevance vs. human selection probability; Bottom: Relevance vs. logit of human selection probability for the monster with the highest relevance predicted by the relevance model.

*Choice of pointing*. For each map across participants, we compute the probability of each monster being selected. This probability can be denoted as $p_{\mathrm{human}}$ . Initially, we put all the monsters from all maps together, treating all monsters independently. We calculate the relevance of each monster $Relevance\left(u|b_{P},b_{H}\right)$ in each map by using Equation 8 and use it to predict the probability of choosing the monster by using Equation 9. $p_{\mathrm{relevance}}\propto \exp\left\{\alpha Relevance\left(u|b_{P},b_{H}\right)\right\}$ . With a fitted α=0.023 , the participants’ choice of pointing can be linearly predicted by a softmax of relevance, as shown in Figure 4. The correlation between $p_{\mathrm{human}}$ and $p_{\mathrm{relevance}}$ is r(40)=0.696,p<.005 . When using $p_{\mathrm{relevance}}$ to linearly predict $p_{\mathrm{human}}$ with the equation $p_{\mathrm{human}}=\beta_{0}+\beta_{1}p_{\mathrm{relevance}}$ , the regression coefficient is ${\hat{\beta }}_{1}=1.302,t\left(40\right)=6.138,p<.001,R^{2}=0.485$ .

Since the selection of all the monsters within the same map are not independent, but mutually exclusive, we then specifically look at the most relevant target within each map. We assume that the higher the relevance, the more likely it is to be picked by the participants. A linear regression analysis shows that the probability of choosing the most relevant monster can be predicted by the exponential of its relevance (Figure 4). In most maps, the monster with the highest relevance is frequently chosen. However, in three maps, no monster has a very high relevance (less than 10), including the monster with the highest relevance. In those maps, we expect the probability of the monster with the highest relevance being chosen to be lower than in other maps, since the most relevant monster is only slightly better than the other two monsters.

We calculate the logit of $p_{\mathrm{human}}$ for the most relevant monster on each map, $logit\left(p_{\mathrm{human}}\right)=\log\frac{p_{\mathrm{human}}+\epsilon }{1-p_{\mathrm{human}}+\epsilon }$ , with a negligibly small hyperparameter arbitrarily chosen as ϵ=0.001 . The correlation between relevance and $logit\left(p_{\mathrm{human}}\right)$ is r(12)=0.830,p<.001 . If we use relevance to linearly predict the logit of $p_{\mathrm{human}}$ with the equation $logit\left(p_{\mathrm{human}}\right)=\beta_{0}+\beta_{1}relevance$ , the regression coefficient is ${\hat{\beta }}_{1}=0.033,t\left(12\right)=5.158,p<.001,R^{2}=0.689$ .

To further analyze the act of pointing to monsters, we study the choice of monsters that fall into the four categories introduced in the stimuli section. Participants chose the monsters off all shortest paths in only 3.93% of the trials. It is very close to 0 as predicted by the relevance model. In the 7 out of 14 maps where the relevance of the most relevant monster is high (>40) , participants chose the most relevant monster in 80.71% of the trials. For the maps where blocking occurs, participants chose the most relevant monster in 80.00% of the trials. In 16.67% of the trials, they chose the second most relevant monster, the blocked one.

*Helpfulness self-rating*. We also examine the participants’ self-rating of their pointing’s helpfulness. When analyzing helpfulness self-rating data, we excluded data from one participant who reported not to correctly understand the rating. It is shown that when the participants pointed to the most relevant monster, they rated the pointing as more useful. We use whether the participant chose the most relevant monster (M=6.87,SD=1.94) or not (M=5.97,SD=2.01) to predict their self-rating of helpfulness and fit a fixed-effect linear model $rating=\beta_{0}+\beta_{1}I\left(highest\;relevance\right)+\alpha_{i}participant_{i}$ . ${\hat{\beta }}_{1}=0.640,p<.001,R^{2}=0.360$ .

*Self-report strategies*. We check the participants’ strategies in the game, which were self-reported after the experiment. As shown in Table 1, 5 out of 11 participants who reported their strategy mentioned that they took the player’s perspective of making decisions, showing the ToM involvement in the signaling process. 9 out of the 11 participants mentioned action prediction and evaluation with phrases such as “shortest path”, indicating their use of paternalistic evaluation of beliefs. Notably, one participant reported forward simulation. “If I feel two points are both important, I will point to one but give myself a low rating. Because I feel pointing to one is not enough, it may also hit the other one.”

**TABLE 1:** Strategies reported by participants in Experiment 1.

Try to interpret my partner’s strategy, and find out the monster which would be most helpful to point out in respond to the partner’s strategy
1. find all possible shortest path from partner’s point of view; 2. signal the monster that blocks the most number of paths
If the monster is right beside the starting point or the goal, I point to that monster. If not, I plan a shortest path myself and see which monster impacts the path the most
If two monsters are next to each other, I’ll choose one of them. If a monster is on the shortest path that the partner is likely to pass, I will point to it and make the partner circumvent to a longer and safer path
Try to think in my partner’s shoes and determine which monster is the most dangerous if I were the partner
Plan a path in my mind as a player and if that path passes through a monster, I click on that monster
Choose three shortest paths that I prefer and see which monster impacts them the most
I tried to use the pointing signal to warn the partner of a direction that could potentially harm its utility the most. It’s a pity that I could not point to an empty space since sometimes pointing to an empty space does a better job on that
If I feel two points are both important, I will point to one but give myself a low rating. Because I feel pointing to one is not enough, it may also hit the other one
I choose one point. If I say my choice is helpful, that means people can avoid the shortest paths through it. If it’s not helpful, then there is another point on the shortest path
I imagine I’m the partner. I will choose one route to the goal that I like best. But this route almost always hits monsters. Back to the perspective of myself, I know the first monster on this route and I will point it out

#### 3.2.5 Discussion

The linear relationship between relevance and the logit of the participants’ choice probability indicates that humans utilize utility-based relevance when deciding which information to communicate. Evidenced by the ratings, humans possess an awareness of the degree of helpfulness associated with each piece of information and thus tend to offer the most beneficial information. The participants’ self-reported strategies clearly show that humans engage in action prediction and evaluation when assessing relevance. Human participants also incorporate counterfactual reasoning, although they do not explicit express it in their self-reflections.

### 3.3 Experiment 2

We flip the role of the human participant and AI in Experiment 1. We assigned participants as the player in our task, while the helper is a rational AI who communicates either with our relevance model or a heuristics model. The heuristic model points to the monster closest to the starting point as measured by Manhattan distance. For example, in Figure 1C, the heuristic model will point to (0, 2). In 10 out of the 28 maps, the heuristic model points to the same monster as the relevance model.

Our goal is to test a) whether a relevance model can contribute more to task performance than a heuristic model and b) whether the help from a relevance model is better received by human participants.

#### 3.3.1 Participants

21 undergraduate students participated in this study and were compensated with course credits. One participant was excluded from the analysis, because all their helpfulness ratings were marked as 1.

#### 3.3.2 Stimuli

The maps were the same as in Experiment 1.

#### 3.3.3 Procedure

Participants entered the experiment room and opened the link to the experiment on a computer. Then, participants went through a practice phase similar to Experiment 1. After the practice phase, the participants started the formal 28 trials, arranged in the same manner as Experiment 1. In each formal trial, participants saw the map without monsters, displayed $8.5\times 8.5{\text{cm}}^{2}$ in the center of the screen. After 3–5 s, a highlight on a cell showed the monster marked by the helper. The participant then controlled the keyboard to navigate the map to the goal. The reward gained by the participants was recorded.

After the participant reached the goal, all the monsters were revealed on the map. A feedback box appeared, showing the participant how many steps they took, how many monsters they ran into, and the score they received. The feedback box also contained a nine-point Likert scale asking the participants to rate the helpfulness of the signal (from 1-not helpful to 9-very helpful). The rating was also recorded for analysis.

#### 3.3.4 Results

Reward. We compare the reward from relevance model and the heuristic model. A paired t-test shows that the participants achieve higher reward when receiving help from the relevance model (M=54.636,SD=17.949) than when receiving help from the heuristic model (M=42.879,SD=12.352) . t(19)=2.761,p<.05 .

Specifically, we compare the reward received in the 18 maps where the relevant helper and the heuristic helper point to different monsters. In these 18 maps, a paired t-test shows that the participants achieve higher reward when receiving help from the relevance model (M=59.111,SD=19.300) than when receiving help from the heuristic model (M=40.178,SD=16.006) . t(19)=3.903,p<.001 .

*Helpfulness rating*. We compare the ratings received from the participants to the relevance model and the heuristic model. A paired t-test shows that the participants give higher ratings to the help from relevance model (M=5.593,SD=1.53) than to the help from the heuristic model (M=5.129,SD=1.47) . t(19)=3.652,p<.005 .

Furthermore, we compare the rating received by participants in the 18 maps where the relevant helper and the heuristic helper point to different monsters. In these 18 maps, a paired t-test shows that the participants give higher ratings to the help from the relevance model (M=5.706,SD=1.697) than to the help from the heuristic model (M=4.983,SD=1.404) . t(19)=3.120,p<.01 .

### 3.4 Experiment 3

Studies have argued that large language models (LLMs) show ToM as well as planning ability, or even navigation (Sap et al., 2022; Guo et al., 2023; Sumers et al., 2023b; Wang et al., 2024; Lin et al., 2024; Park et al., 2023), by turning planning games into a language game. Since we have a task demanding a coordination of mind reasoning, we are interested in how GPT may perform. We turn our task in Experiment 1 to a language game and use the state-of-the-art large language model (LLM) GPT-4 (OpenAI et al., 2023) as a participant. GPT-4 was provided with a description of the game and was asked to choose one of the three monsters to point to.

#### 3.4.1 Stimuli

An example of the prompt we provided is: “You are playing a two-person game. A player navigates a 5×5 grid map to a goal. In the map, there are three monsters invisible to the player. The player can see the location of the goal. When the player reaches the goal, the game ends. The player can also see the walls on the map that they cannot go through. The walls are between 2 cells in the map. The player going one step costs 1 dollars. Reaching the goal grants 100 dollars and running into each monster costs 100 dollars. We want to get the highest reward. You play as a helper who can inform the player of the location of only one monster. Do you understand the game?”

After GPT-4 repeated the correct rules, we entered the second prompt: “If the player starts from (0, 0), the goal is (2, 4), no walls are in the map, the monsters are at (1, 2), (2, 0), and (4, 2). Which monster will you tell the player?”

We recorded GPT-4’s responses and compared them with the human data collected from Experiment 1.

#### 3.4.2 Results

Choice of pointing. We compared GPT-4’s choices with the human participants’ from Experiment 1.

42.1% of human participants’ choices are consistent with the choices from GPT-4. In comparison, 58.2% of human participants’ choices are consistent with the choices from our relevance model. Humans’ choices are more consistent with our relevance model than GPT-4’s prediction, z=−5.379,p<.001 .

**FIGURE 5:**
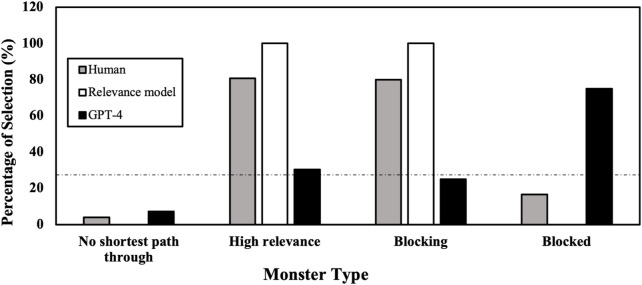
4 of the maps used in the experiments. **(A)** Orange monster high in relevance while green monster low in relevance; **(B)** Red monster high in relevance while orange and green monsters low in relevance; **(C)** Orange monster blocked by red monster; **(D)** Red monster blocked by green monster.

We analyzed the pointing to each monster in the four categories in more detail (Figure 5). In 13 of the 14 maps, GPT-4 will not point to the monster off any shortest path.

Is GPT-4 looking at the most valuable monster target? In the seven maps where one monster is very relevant (the calculated relevance of a monster is over 40), GPT-4 only chose the same monster as humans do in 30.4% of the trials, far from the consistency between the relevance model and human participants (80.7%) . This shows that GPT is aware of what the shortest paths are, and that monsters not on any shortest paths is not relevant.

In the maps where one monster blocks another, GPT-4 chose the most relevant monster with a probability of 25% . In the other 75% of the trials, it chose the blocked monster. For example, in Figure 1D, where the red monster is blocked by the green one. GPT-4 points to the red monster, which is rarely consistent with human choice. As we speculated when we designed the maps, even though recent studies shows evidence for LLMs using counterfactual reasoning (Zhang et al., 2024), their counterfactual reasoning cannot not be spontaneously utilized.

Self-report strategies. GPT-4 also provided legit and reasonable strategies that were similar to the human participant reports in Experiment 1. An example is shown in Table 2 The answers clearly involve action prediction and evaluation. GPT-4 assumes one trajectory for the player and points to a monster on the trajectory. However, it does not consider all of the player’s possible policies. We see human participants report this strategy too, but their choices of pointing to monsters are different from GPT-4.

**TABLE 2:** Examples of strategies reported by GPT-4 in Experiment 3.

Given the new configuration, the hunter’s most efficient path avoiding the walls would be: Move left 2 times to (2, 0) Move up 2 times to (2, 2) Move left 2 times to (0, 2) Move up 2 times to (0, 4) Move right 2 times to (2, 4), the goal Following this path, the monster at position (2, 0) is the one directly in the hunter’s way. Therefore, you should warn the hunter about the monster at (2, 0)
Given the walls and monsters’ positions, the best monster to reveal to the hunter would be the one at position (3, 1) Here’s why: Monster at (3, 1): This monster is directly on the hunter’s potential path towards the goal. Given the walls, the hunter might decide to move left and then up, colliding with this monster Monster at (3, 2): This monster is also on a potential path to the goal, but the wall between (3, 2) and (2, 2) will prevent the hunter from moving in this direction Monster at (0, 4): This monster is far from the hunter’s starting position and the goal. It’s unlikely the hunter would move this far to the left, so revealing this monster would not provide a significant benefit The optimal path, if the monster at (3, 1) is revealed, would be: The hunter starts from (4, 0), moves up to (4, 1), then moves up to (4, 2), then moves left to (3, 2), moves up to (3, 3), then moves left to (2, 3), and finally moves up to the goal at (2, 4) This path avoids all monsters and costs $7. So, the monster at (3, 1) is the best choice to reveal to the hunter

#### 3.4.3 Discussion

Overall in the experiment, GPT-4 performs better than chance level, indicating that it owns some capacity needed to solve the game. However, the consistency of GPT-4’s choices with human participants is lower than the consistency of relevance model with participants, indicating that they fall short in planning or ToM ability, compared with human level. Surprisingly, we found that the report of GPT is very similar to human self-report. GPT-4 encompasses the concepts of action prediction and action evaluation, showing a certain level of capacity for ToM and planning. However, GPT-4 is not using the action prediction and action evaluation components to make choices, as humans do. It is more likely that GPT-4 just mimics human thinking processes on a surface level.

## 4 General discussion

Through our experiments, we present concrete evidence that impromptu human communication within cooperative contexts aligns with the principle of utility-based relevance. In our first experiment, we observe that the choices made by human participants regarding which monster to point to can be accurately predicted by our relevance model. Notably, the consistency between the participants’ choices and the model’s predictions remains high across various types of monsters, including those with very low relevance, very high relevance, blocked paths, and those blocking paths.

These findings suggest that when individuals decide what information to convey, they understand the relevance associated with each piece of information. Then they rationally choose the relevant ones. RSA proposes that each utterance in communication carries a utility based on how it alters the listener’s belief towards the true belief in a reference game (Frank and Goodman, 2012). In our experiment, we expand the concept of utility to relevance: the utility of a communicative signal within a task can be defined by how it enhances the listener’s performance. The findings are consistent with recent studies on relevance (Sumers et al., 2021; Jiang et al., 2022; Jiang et al., 2021). This seemingly simple utility calculation indicates that humans maintain a representation and plan in the physical world, which largely influences their communication (Friston et al., 2021).

In Experiment 2, we observe that communication generated by AI integrated with a relevance model yields enhanced performance and receives higher ratings in terms of perceived helpfulness. We aspire that our model can serve as a source of inspiration for designing AI systems that offer both impromptu and efficient interactions with humans.

An AI system equipped with a relevance model only requires planning capacity grounded in decision-making theory to effectively provide concise assistance to humans. This approach could potentially alleviate the intensive training needed for AI to communicate in human natural language or develop a new language (Lazaridou and Baroni, 2020; Havrylov and Titov, 2017). Additionally, by answering the question of what to communicate (Roth et al., 2006), an AI agent may circumvent the necessity of sharing its entire observation from the sensor (Reddy et al., 2021) with humans. Instead, it can pick the most relevant information in the observation to avoid excess information overload in the cases of large observations, such as images or videos. A recent example is the improvement in human-robot joint task performance by communicating goals in (Le Guillou et al., 2023), which is highly relevant in cooperative tasks (Tang et al., 2022).

The strategy descriptions from human participants in Experiment 1 and GPT-4 in Experiment 3 both reflect elements of action prediction and action evaluation. It may leave the impression that GPT-4 possesses ToM abilities similar to humans (Sap et al., 2022). However, such similarity in language-described strategy is not supported by their choice of signals. In other words, while GPT-4 may sound like a human, it does not always behave like one (Mahowald et al., 2023). Human decisions across all four monster types align well with action prediction and action evaluation as modeled by the relevance framework, but the behavior of GPT-4 only agrees with the ToM model on one type. This discrepancy might stem from gaps between human reports and human behavior: Since GPT-4 is trained from language data, it may fail to learn certain reasoning processes if they are not frequently articulated in human language. Supporting this hypothesis, in Experiment 1, human choices reflect counterfactual reasoning consistent with the prediction of our relevance model, yet this reasoning is rarely made explicit in the participants’ verbal reports. This suggests the need for more direct evaluations of behavior of LLMs as agents: other than its language output as a chatbot, we should also focus on its actions.

In the thriving trend of reinforcement learning with human feedback (Ouyang et al., 2022), it is pivotal to appreciate the value of incorporating behavioral data into model training and conducting data analysis guided by theories in cognitive science. We hope to inspire more interdisciplinary research combining ToM and AI models like POMDP.

## Data Availability

The datasets analyzed for this study can be found in the Github repository: https://github.com/kaiwenj/relevanceCommunication.
